# Supplementary material for: Chemotherapy effectiveness in trial-underrepresented groups with early breast cancer: A retrospective cohort study
Source: PLoS Med. 2019 Dec 31;16(12):e1003006. doi: 10.1371/journal.pmed.1003006 (PMC6938317; doi:10.1371/journal.pmed.1003006)
Supplement: S1 Table — (DOCX) [file pmed.1003006.s002.docx]

|  | SCR 2001-2015 | | SCR 2001-1015 | |
| --- | --- | --- | --- | --- |
|  | Over 70 | | High Comorb. | |
|  | Number | (%) | Number | (%) |
| Nodes 0 | 6199 | 64.2 | 5730 | 71.9 |
| Nodes 1 | 1370 | 14.2 | 1039 | 13 |
| Nodes 2-4 | 1087 | 11.3 | 736 | 9.2 |
| Nodes 5-9 | 505 | 5.2 | 224 | 2.8 |
| Nodes 10+ | 421 | 4.4 | 203 | 2.5 |
| Grade I | 1078 | 11.2 | 1589 | 19.9 |
| II | 4970 | 51.5 | 3947 | 49.6 |
| III | 3552 | 36.8 | 2388 | 30 |
| ER- | 1611 | 16.7 | 1023 | 12.8 |
| ER+ | 8042 | 83.3 | 6942 | 87.2 |
| Chemotherapy | 981 | 10.2 | 2558 | 32.1 |
| Endocrine therapy | 7428 | 77 | 6174 | 77.5 |
| Combined chemoendocrine | 485 | 5 | 1564 | 19.6 |
| Screen detected | 1898 | 19.7 | 5287 | 66.4 |
| Symptomatic | 7739 | 80.2 | 2659 | 33.4 |
| Charlson >= 1 | 875 | 9.1 | 1655 | 20.8 |
| Charlson = 0 | 8778 | 90.9 | 6310 | 79.2 |
