# Supplementary material for: Chemotherapy effectiveness in trial-underrepresented groups with early breast cancer: A retrospective cohort study
Source: PLoS Med. 2019 Dec 31;16(12):e1003006. doi: 10.1371/journal.pmed.1003006 (PMC6938317; doi:10.1371/journal.pmed.1003006)
Supplement: S2 Table — (DOCX) [file pmed.1003006.s003.docx]

| Variable | Mean untreated | Mean treated | P NH: m1=m2 | SMD |
| --- | --- | --- | --- | --- |
| Women aged over 70 | | | | |
| Age | 73.29 | 73 | 0.034 | 0.114 |
| PREDICT 10y mort. | 0.62 | 0.61 | 0.164 | 0.075 |
| Screen detected | 0.16 | 0.17 | 0.665 | -0.023 |
| No. Positive nodes | 3.66 | 3.64 | 0.942 | 0.004 |
| Tumour size | 30.9 | 30.34 | 0.548 | 0.032 |
| grade 2 | 0.25 | 0.3 | 0.03 | -0.117 |
| grade 3 | 0.74 | 0.69 | 0.065 | 0.099 |
| ER + | 0.6 | 0.61 | 0.826 | -0.012 |
| log inpatient days | 0.54 | 0.57 | 0.664 | -0.023 |
| log outpatient apts | 1.25 | 1.26 | 0.981 | -0.001 |
| Charlson (0/1) | 0 | 0 | 0 | 0 |
| SIMD | 3.07 | 3.08 | 0.832 | -0.011 |
| hormone therapy | 0.54 | 0.54 | 0.914 | 0.006 |
| radiotherapy | 0.71 | 0.7 | 0.639 | 0.025 |
| Women with high comorbidity | | | | |
| Age | 59.03 | 58.64 | 0.07 | 0.038 |
| PREDICT 10y mort. | 0.33 | 0.32 | 0.036 | 0.044 |
| Screen detected | 0.33 | 0.36 | 0.002 | -0.063 |
| No. Positive nodes | 1.45 | 1.43 | 0.725 | 0.007 |
| Tumour size | 22.85 | 23.13 | 0.308 | -0.021 |
| grade 2 | 0.44 | 0.46 | 0.038 | -0.043 |
| grade 3 | 0.51 | 0.48 | 0.003 | 0.063 |
| ER + | 0.81 | 0.82 | 0.232 | -0.025 |
| log inpatient days | 0.38 | 0.38 | 0.7 | 0.008 |
| log outpatient apts | 1.13 | 1.13 | 0.746 | 0.007 |
| Charlson (0/1) | 0.06 | 0.06 | 0.48 | 0.015 |
| SIMD | 3.05 | 3.07 | 0.476 | -0.015 |
| hormone therapy | 0.73 | 0.73 | 0.778 | -0.006 |
| radiotherapy | 0.69 | 0.68 | 0.293 | 0.022 |

SMD: Standardised mean difference, difference in means of two groups divided by the standard deviation, assuming independence: $\frac{\mu_{2}-\mu_{1}}{\sqrt{{\sigma_{2}}^{2}+{\sigma_{1}}^{2}}}$

P NH: m1=m2: P value for a test of the null hypothesis of equal mean for treated and untreated groups calculated using a two sample t test.
