# Supplementary material for: Chemotherapy effectiveness in trial-underrepresented groups with early breast cancer: A retrospective cohort study
Source: PLoS Med. 2019 Dec 31;16(12):e1003006. doi: 10.1371/journal.pmed.1003006 (PMC6938317; doi:10.1371/journal.pmed.1003006)
Supplement: S3 Table — (DOCX) [file pmed.1003006.s004.docx]

| Specification | Coefficient | P | 95% CI  lower | 95% CI  upper |
| --- | --- | --- | --- | --- |
| Women aged over 70 | | | | |
| IV1 All-cause | 0.486 | <0.001 | 0.367 | 0.604 |
| IV2 All-cause | 0.135 | 0.006 | 0.04 | 0.231 |
| IV 1 BC | 0.409 | <0.001 | 0.29 | 0.527 |
| IV 2 BC | 0.131 | 0.007 | 0.036 | 0.225 |
| Women with high comorbidity | | | | |
| IV1 All-cause | 0.411 | <0.001 | 0.261 | 0.561 |
| IV2 All-cause | 0.102 | 0.012 | 0.023 | 0.182 |
| IV1 BC | 0.397 | <0.001 | 0.246 | 0.547 |
| IV2 BC | 0.105 | 0.01 | 0.025 | 0.185 |
