# Supplementary material for: Chemotherapy effectiveness in trial-underrepresented groups with early breast cancer: A retrospective cohort study
Source: PLoS Med. 2019 Dec 31;16(12):e1003006. doi: 10.1371/journal.pmed.1003006 (PMC6938317; doi:10.1371/journal.pmed.1003006)
Supplement: S4 Table — (DOCX) [file pmed.1003006.s005.docx]

| PSM specification | Breast Cancer | | | | | All | | | |
| --- | --- | --- | --- | --- | --- | --- | --- | --- | --- |
|  | N | Deaths | HR | 95% CI lower | 95% CI upper | Deaths | HR | 95% CI lower | 95% CI upper |
| Over 70 and no comorbidity | | | | | | | | | |
| NN 1:1 no replacement, within calliper | 1302 | 407 | 0.79 | 0.66 | 0.99 | 565 | 0.72 | 0.61 | 0.85 |
