# Supplementary material for: Chemotherapy effectiveness in trial-underrepresented groups with early breast cancer: A retrospective cohort study
Source: PLoS Med. 2019 Dec 31;16(12):e1003006. doi: 10.1371/journal.pmed.1003006 (PMC6938317; doi:10.1371/journal.pmed.1003006)
Supplement: S5 Table — (DOCX) [file pmed.1003006.s006.docx]

| Specification | Coefficient | P value | 95% CI lower | 95% CI upper |
| --- | --- | --- | --- | --- |
| IV1 All-cause | 0.486 | <0.001 | 0.341 | 0.63 |
| IV2 All-cause | 0.101 | 0.082 | -0.013 | 0.215 |
| IV 1 BC | 0.408 | <0.001 | 0.263 | 0.553 |
| IV 2 BC | 0.101 | 0.078 | -0.011 | 0.214 |
