# Supplementary material for: Chemotherapy effectiveness in trial-underrepresented groups with early breast cancer: A retrospective cohort study
Source: PLoS Med. 2019 Dec 31;16(12):e1003006. doi: 10.1371/journal.pmed.1003006 (PMC6938317; doi:10.1371/journal.pmed.1003006)
Supplement: S6 Table — (DOCX) [file pmed.1003006.s007.docx]

| Specification / Outcome | HR | 95% CI lower | 95% CI upper |
| --- | --- | --- | --- |
| IV1 all | 0.65 | 0.5 | 0.81 |
| IV2 all | 0.67 | 0.51 | 0.83 |
| IV1 BC | 0.6 | 0.43 | 0.81 |
| IV2 BC | 0.6 | 0.43 | 0.83 |
