# Supplementary material for: Chemotherapy effectiveness in trial-underrepresented groups with early breast cancer: A retrospective cohort study
Source: PLoS Med. 2019 Dec 31;16(12):e1003006. doi: 10.1371/journal.pmed.1003006 (PMC6938317; doi:10.1371/journal.pmed.1003006)
Supplement: S1 Text — (DOCX) [file pmed.1003006.s008.docx]

**Plan and methods**

The research project will be divided into three phases. The first phase will consist of preparing the data for analysis. Then, the external validity of the scoring tools will be assessed and the feasibility of applying a RD design will be determined. Finally, estimates of treatment effects from the RD design will be compared to treatment effects extracted from the literature.

***Data preparation (months 1-6)***

Patients with a new diagnosis of invasive breast cancer treated with curative surgical resection between 2001 and 2011 will be identified from the Scottish Cancer Registry providing a sample size greater than 24,000 patients (estimated from Scottish Cancer Networks Audit data). A data extract from the registry (SMR06) containing staging and initial treatment information is already linked to hospital discharge data (SMR01) and mortality data and will be provided by Information Services Division (ISD) and stored for analysis within the National Services Scotland Safe Haven. Approval will be sought from the Public Benefit Privacy Panel.

Information required to calculate the scores central to the implementation of the empirical strategy are shown in table A. The Scottish Cancer Registry has been shown to be global leader in the completeness and reliability of data, with few discrepancies compared to other nations. Based on pilot data we anticipate missing data in surgically treated patients being below 5% for the majority of required variables. During this phase of the project, data-fields for a sample of the population will be cross-validated with Network Audit teams which have 100% source data verification since 2008 to provide a more robust quantification of missing data. Co-morbidity scores will be calculated from pre-diagnosis inpatient diagnoses (SMR01) using methods previously validated by ISD. In all analyses described below, the sensitivity of results to missing data will be assessed by comparing results from: 1) a complete case analysis and 2) an analysis involving multiple imputation.

***Assessment of score-based treatment assignment mechanism (months 9-11):***

A crucial step for implementing the RD method is the assessment of the treatment assignment mechanism, i.e. whether the probability of receiving the treatment changes sharply around score thresholds, and whether patients with scores close to the threshold are observationally similar. We will first describe the distribution of predicted 10-year survival benefit from chemotherapy computed using NHS Predict and Adjuvant! Online and identify potential clusters around the decision thresholds (i.e. 3% and 5%). Figure 1 illustrates the three main situations encountered when analysing data anticipated to be suitable to RD. Panel A illustrates the “ideal” case, where there is strict adherence to the cut-off rule (“sharp” RD design). In such case, treatment allocation is clear around the threshold. Conversely, panel B illustrates a case where many individuals with values above the cut-off are not treated and others below the cut-off are treated (“fuzzy” RD design). Panel C reflects a case where there is no discontinuity at the cut-off and where RD would not be appropriate.

[Figure 1 was example of RD design figures]

Given the weight of clinician and patient preferences on the decision to give chemotherapy, and the number of potential unobserved treatment determinants, the data for our study will likely correspond to a “fuzzy” RD design. However, this will have to be formally determined by analysing the probability of receiving chemotherapy treatment as a function of the score, and carefully investigating changes at the decision thresholds. The example figure below illustrates one possible shape for the probability to be treated around the threshold of a risk score8. In this particular case, we observe a sharp change in the probability to be treated around the threshold (0.2 in this case), which is what is required for identification.

Such analysis, and the implementation of RD more generally, requires sufficient sample size as the effects of interests are identified in a sub-population of patients with scores in the vicinity of the thresholds. Based on Lothian audit data, currently 28% of surgically treated patients (c. 7000 patients) fall *within* the 3-5% marginal predicted benefit category, which ensures sufficient sample size to implement the RD design and base the estimation of local average treatment effects on a reasonably large sample size.

As a last step to assess the internal validity of the RD design, we will assess covariate balance around the threshold using patient characteristics not included in the scoring process, such as comorbidity and deprivation9. Practically, we will perform a series of statistical tests to assess the difference in these characteristics between groups of patients based on their calculated risk score. This will be conducted for a range of “bandwidths” around the threshold (i.e. 2-3% v.s. 3-4%; 1-3% vs. 3-5%, etc.). These preliminary analyses will allow us to assess the likely biases influencing the assignment mechanism and to make a decision on the most appropriate threshold value to use. In addition to the mean estimates, we will verify whether the distribution of these variables are smooth around the threshold, as a key assumption of the RD design is that nothing else other than the probability of being treated changes sharply around the threshold.

***Treatment effect estimates and assessment of bias (months 12-15):***

If the validity of applying RD to these data is demonstrated, the design will be implemented in the full sample of 24,000 breast cancer patients to obtain reference treatment effect estimates. Formal criteria for progressing with this stage will depend on the feasibility of using the RD design in this setting and will be ratified by our Advisory Group. Based on the work of Moscoe et al 2015 they will depend on demonstration that: (i) the assignment variable is measured and reported continuously, (ii) the outcome variables are observed for all patients, independent of whether they were assigned the treatment or not and, (iii) precise information on how treatment is assigned to patients is available to determine whether the design is ‘‘sharp’’ or ‘‘fuzzy.’’ Table B provides an overview of the main elements of the “fuzzy” RD design using the formal notation of the causal inference literature. Briefly, our setting resembles that of an instrumental variable strategy where the cut-off values are associated with discrete changes in the probability to receive the treatment (i.e. impact of the score cut-off on expected outcomes weighted by the impact of the cut-off on the probability to be treated). We will focus on overall survival and breast cancer-specific survival as outcomes at 5 and 10 years and will assess the sensitivity of the results to methodological choices (i.e. the “bandwidth” around the threshold and functional form). We will then compare RCT-based treatment effects to those obtained from RD in a trial-eligible sub-population19. Relevant RCT-based treatment effects will be extracted from the literature along with details on the population in which these effects were obtained. When possible, treatment effects obtained in subgroups of patients will be extracted (by age group, gender, etc.). The RD design will then be applied in a matched sample of patients from the registry data. The matching could be based on a single characteristic (e.g. restriction to patients in a particular age group) or involve multiple criteria. We will use the procedure described by Altman and Bland20 to compare the treatment effects estimates obtained with the RD design to those extracted from the literature. This will allow quantifying the potential bias of RD as compared to RCT and assessing whether RD can be implemented routinely for the assessment of the real-world effectiveness of chemotherapy. If the estimates derived from RD in the RCT populations match within 10%, we will finally apply RD methods to subgroups of individuals for whom RCT evidence is lacking, such as older patients and those with co-morbidities or high social deprivation to provide a first empirical estimate for these groups.
