# Supplementary material for: Chemotherapy effectiveness in trial-underrepresented groups with early breast cancer: A retrospective cohort study
Source: PLoS Med. 2019 Dec 31;16(12):e1003006. doi: 10.1371/journal.pmed.1003006 (PMC6938317; doi:10.1371/journal.pmed.1003006)
Supplement: S2 Text — (DOCX) [file pmed.1003006.s009.docx]

**Analysis Protocol**

The analysis protocol for this study was to follow the analysis in (1) conditional on that analysis determining which methods for real world evidence were feasible in these data, and could provide estimates of treatment effect concordant with evidence from randomised data for breast-cancer specific and/or all-cause mortality. This general strategy for analysis was decided prior to getting access to the data. PSM with the approach specified below and two IV analyses were finally included on the basis of the strategy. No more detailed protocol or statistical analysis was written prior to access to the data. No alterations were made to the analysis following knowledge of outcomes in these specific patient groups.

The analysis was a complete case analysis. This was considered appropriate based on previous analysis using these data for other purposes and which compared multiple imputation and complete case analysis, finding little difference in PREDICT prognostic accuracy including or not including cases with missing data (2). The period of follow-up for each patient was calculated from date of diagnosis until either date of death, embarkation from UK (very few cases) or 1^st^ April 2018. All statistical analyses were carried out using Stata version 14 software.

**Propensity Score Matching**

Propensity scores were generated by probit regression. The dependent variable was a binary indicator of adjuvant chemotherapy use. Explanatory variables include in the model were: PREDICT 10-year probability of mortality, age at diagnosis, number of positive lymph nodes, pathological tumour size, tumour histological grade, mode of detection, ER status, Her2 status, hormone therapy use, radiotherapy use, year of diagnosis, Scottish Index of Multiple Deprivation (SIMD) quintile, Charlson comorbidity status, log total inpatient bed days and log total outpatient visits (5 years prior to diagnosis). In the analysis with breast cancer death as the outcome PREDICT 10-year probability of breast cancer mortality was used in instead of PREDICT 10-year probability of mortality. Interactions of other clinical prognostic factors with ER status were also included. Predicted probabilities of chemotherapy use – the propensity score - for each individual observation were estimated from this regression model.

Matching of treated and non-treated observations used nearest-neighbour matching on the propensity scores. The preferred matching methods was selected based on considerations reported in (1). This was 1:1 matching without replacement within calipers set to 0.25 standard deviations of the logit of the propensity score, as suggested by (3). Matching was restricted to observations within the region of common support (only treated observations with propensity scores between the maximum and minimum scores in the non-treated observations were included). Furthermore, the sample was trimmed to observations ranging from a propensity score of 0.05 to 0.95 prior to matching.

To examine the quality of the matches achieved by each matching method, baseline covariate balance in the matched treated and non-treated samples was assessed by comparing the means or proportions of baseline covariates.

Cox regression was used to estimate hazard ratios for chemotherapy in the matched samples. Proportional hazard was assumed based on reported results from the trial meta-analysis which found no evidence against this assumption (4), it was assumed this would also hold in the RWD setting. Confidence intervals were calculated by a simple bootstrap of individual cases with 1000 iterations.

**Instrumental Variables Analysis**

In the preceding study in the trial representative population (1) a number of candidate instruments were considered and two were selected to be used in treatment effect estimation: PREDICT benefit score (IV 1) and the PREDICT benefit score interaction with a post 2010 dummy variable (IV 2). See the supplemental appendix of (1) for details of all instruments considered and reasons for exclusion or infeasibility of those not selected. There are potential advantages and disadvantages of each selected instrument. IV1 is likely to be more efficient (greater statistical power) but with greater potential bias while IV2 is likely to be less efficient but has less potential for bias. The validity of IV1 relies on the benefit score being independent from expected survival conditional on the prognostic score and chemotherapy use. As benefit score is the difference in expected survival with and without chemotherapy this should be the case by construction, however it requires the prognostic model to be well calibrated. IV2 exploits the introduction of the PREDICT online tool in 2010, in this case validity rests on the assumption that the ability to access this information influenced the decisions made across at least part of the range of PREDICT benefit scores.

Two-stage residual inclusion (2SRI) was used to estimate adjuvant chemotherapy treatment effects (5). This method can provide consistent estimates of the treatment effect in situations with non-linear first and second stage regressions (due to limited dependent variables). The first stage was estimated by logistic regression and the second stage was estimated by Cox regression. Confidence intervals were calculated by a simple bootstrap of individual cases with 1000 iterations. A Wald test was used to assess the strength of the proposed instruments in the first stage regression. The assumptions of independence of the instrument from unmeasured confounding variables and that the instrument does not affect outcomes expect through the effect on treatment status cannot be empirically tested. A judgement on their validity in this case must be reached by consideration of theory and the understanding of the decision making process.

## References

1. Gray E, Marti J, Brewster DH, Wyatt JC, Piaget-Rossel R, Hall PS. Real-world evidence was feasible for estimating effectiveness of chemotherapy in breast cancer: a cohort study. J Clin Epidemiol. 2019 May;109:125–32.

2. Gray E, Marti J, Brewster DH, Wyatt JC, Hall PS. Independent validation of the PREDICT breast cancer prognosis prediction tool in 45,789 patients using Scottish Cancer Registry data. Br J Cancer. 2018 Oct 17;119(7):808–14.

3. Austin PC. Optimal caliper widths for propensity-score matching when estimating differences in means and differences in proportions in observational studies. Pharm Stat [Internet]. 2011;10(2):150–61. Available from: %3CGo

4. Early Breast Cancer Trialists’ Collaborative Group. Comparisons between different polychemotherapy regimens for early breast cancer: meta-analyses of long-term outcome among 100 000 women in 123 randomised trials. Lancet. 2012;379(9814):432–44.

5. Terza J V, Basu A, Rathouz PJ. Two-stage residual inclusion estimation: addressing endogeneity in health econometric modeling. J Heal Econ. 2008;27(3):531–43.
