# Supplementary material for: Chemotherapy effectiveness in trial-underrepresented groups with early breast cancer: A retrospective cohort study
Source: PLoS Med. 2019 Dec 31;16(12):e1003006. doi: 10.1371/journal.pmed.1003006 (PMC6938317; doi:10.1371/journal.pmed.1003006)
Supplement: Attachment — Submitted filename: Requests from Editors 2 responses.docx [file pmed.1003006.s010.docx]

| Title- please remove "real world evidence" | This has been removed |
| --- | --- |
| DAS- please revise to “anonymously” Please to provide a url and accession number needed to apply for data access | DAS has been revised. |
| Did your study have a prospective protocol or analysis plan? Please state this (either way) early in the Methods section.  a) If a prospective analysis plan (from your funding proposal, IRB or other ethics committee submission, study protocol, or other planning document written before analyzing the data) was used in designing the study, please include the relevant prospectively written document with your revised manuscript as a Supporting Information file to be published alongside your study, and cite it in the Methods section. A legend for this file should be included at the end of your manuscript.  b) If no such document exists, please make sure that the Methods section transparently describes when analyses were planned, and when/why any data-driven changes to analyses took place.  c) In either case, changes in the analysis—including those made in response to peer review comments—should be identified as such in the Methods section of the paper, with rationale."  At this stage, we ask that you include a short, non-technical Author Summary of your research to make findings accessible to a wide audience that includes both scientists and non-scientists. The Author Summary should immediately follow the Abstract in your revised manuscript. This text is subject to editorial change and should be distinct from the scientific abstract. Please see our author guidelines for more information: <https://journals.plos.org/plosmedicine/s/revising-your-manuscript#loc-author-summary> | There was an overall prospective analysis plan for the project in the funding proposal. This has now been included in the supplementary material and cited in the text. In addition, the cited study (JCE) directly determined the more detailed analysis plan for this study. This was partially a data-driven process although importantly not driven by knowledge of outcomes. The analysis of the previously published study strictly preceded the analysis for this study and used a non-overlapping patient population. These details have been clarified in the methods section. The funding proposal has been added to the supplemental material and is cited in the text. A legend is also included at the end of the manuscript.  A change in the analysis introduced at the review stage was the addition of the application of results in a recalibrated prognostic model. The text has been amended to state that this change was made at review stage in response to comments about clinical interpretation (p7).  An author summary section has been added to the manuscript. |
| Abstract- Background refers to early stage breast cancer and the methods and findings section refers to primary. If both are the same, could we use consistent language. | Primary was used here because the record extraction from the registry is to first get all primary tumours of the breast then limit to early stage breast cancers. To simplify we have changed primary to early. |
| Square brackets for all references | This change has been made. |
| Footnotes- should be incorporated into main body of text | All footnotes have been added to the main text. Some with minor editing to improve flow and make concise. P6, P7, P8. |
| Discussion lines 22-24 this needs to be revised and toned down since the introduction states problems with recruitment of women over 70 were due to physicians and patients | This is a good point. This section has been revised to make clear that this would also depend on patient’s perceptions of the risks and benefits and ultimately their willingness to participate. |
| Discussion requires a subsection outlining the limitations of the study | The discussion has been restructured so that limitations are presented in a headed subsection. Other headed subsections have been created fit this format. |
| Discussion section requires toning down of the conclusions as this is a retrospective cohort study not a trial. Please provide a brief explanation of how this study design allows you to infer the "effectiveness" versus "efficacy" of chemotherapy. | We certainly want to ensure that the findings of this study are not misinterpreted as a randomised trial rather than as observational data. To ensure this in all parts of the discussion that mention the main finding we always include ‘observational’ in the sentence stating the conclusion. This is followed by the caveat that causal interpretation of observational data requires additional assumptions. In the first sentence of the discussion (p10) we have replaced “these results” with “these observational data”. The following sentence has been rephrased to highlight that interpretation depends on whether one believes that the assumptions required for unbiased causal inference are being met sufficiently in this case. In the concluding section (P13, final paragraph) we believe this has already been quite carefully worded, using “observational data” and “should be interpreted with caution…”.  A sentence has been added to the strengths section of the discussion to highlight how this study relates to the idea of effectiveness rather than efficacy. |
| RECORD checklist should be provided as a stand-alone SI file and page numbers should be removed as they are likely to change during publication | Record checklist has been uploaded as a stand-alone and page numbers removed. |
| Reviewer #3: I am comfortable with the responses and revisions. However, I am slightly confused by the new analyses reporting predicted survival benefits (Tables 3 and 4). In Table 3, the header of columns 3 and 4 state "Mean predicted 10-year survival ... (%)". Are these numbers showing the proportion of women expected to survive at 10 years? If so, I believe the word "mean" should not be there. If it is indeed a mean/average, then I do not understand what these numbers represent. For transparency, I would suggest indicating that these analyses were done post-hoc. The authors might add that they were conducted in response to the initial peer-review.  -Laurent Billot | Yes, this was an error, the headings of table 3 should have been the same as those for table 4. This has been corrected. “Mean predicted survival” is indeed a poor phrase, it referred to taking the mean of the individual predictions, this is equivalent to the predicted survival for the group.  In the methods section a statement has been added that this analysis was in response to peer review comments. Thank you. |
